# Sensitivity of the circadian system to evening bright light in preschool‐age children

**DOI:** 10.14814/phy2.13617

**Published:** 2018-03-04

**Authors:** Lameese D. Akacem, Kenneth P. Wright, Monique K. LeBourgeois

**Affiliations:** ^1^ Sleep and Development Laboratory Department of Integrative Physiology University of Colorado Boulder Boulder Colorado; ^2^ Sleep and Chronobiology Laboratory Department of Integrative Physiology University of Colorado Boulder Boulder Colorado

**Keywords:** Circadian, light, melatonin suppression, preschool children, sleep

## Abstract

Although the light‐induced melatonin suppression response is well characterized in adults, studies examining the dynamics of this effect in children are scarce. The purpose of this study was to quantify the magnitude of evening light‐induced melatonin suppression in preschool‐age children. Healthy children (*n* = 10; 7 females; 4.3 ± 1.1 years) participated in a 7‐day protocol. On days 1–5, children followed a strict sleep schedule. On day 6, children entered a dim light environment (<15 lux) for 1‐h before providing salivary samples every 20‐ to 30‐min from the afternoon until 50‐min after scheduled bedtime. On day 7, subjects remained in dim light conditions until 1‐h before bedtime, at which time they were exposed to a bright light stimulus (~1000 lux) for 1‐h and then re‐entered dim light conditions. Saliva samples were obtained before, during, and after bright light exposure and were time anchored to samples taken the previous evening. We found robust melatonin suppression (87.6 ± 10.0%) in response to the bright light stimulus. Melatonin levels remained attenuated for 50‐min after termination of the light stimulus (*P* < 0.008). Furthermore, melatonin levels did not return to 50% of those observed in the dim light condition 50‐min after the light exposure for 7/10 children. Our findings demonstrate a robust light‐induced melatonin suppression response in preschool‐age children. These findings have implications for understanding the role of evening light exposure in the development of evening settling difficulties and may serve as experimental evidence to support recommendations regarding light exposure and sleep hygiene practices in early childhood.

## Introduction

The human circadian clock, localized to the suprachiasmatic nucleus (SCN) of the anterior hypothalamus, is strongly influenced by light (Czeisler et al. 1981; Ralph et al. 1990; Duffy and Wright 2005). Information on the light/dark cycle is communicated to the circadian clock through direct input from retinal photoreceptors via the retinohypothalamic tract (Moore and Card 1985; Johnson et al. 1988). In response to activation by light, terminals of the retinohypothalamic tract release glutamate on to the SCN (Ebling 1996), which in turn influences outputs of the clock including the sleep promoting hormone melatonin (Gastel et al. 1998).

Secretion of melatonin is regulated by a multisynaptic pathway originating from the SCN and terminating at the pineal gland (Moore and Klein 1974; Teclemariam‐Mesbah et al. 1999; Kalsbeek et al. 2006). This pathway begins with efferent projections from the SCN to the paraventricular nucleus (PVN), which then sends descending projections to the intermediolateral cell column (IML) of the spinal cord. Preganglionic sympathetic neurons then project to the superior cervical ganglion (SCG). This synaptic pathway terminates with postganglionic sympathetic nerve fibers that project onto the pineal gland (Borjigin et al. 1999). During the biological night these postganglionic fibers release norepinephrine onto the pineal gland which stimulates the secretion of melatonin by binding to adrenergic receptors (Drijfhout et al. 1996a,b). Exposure to light during the biological night, however, inhibits norepinephrine release and acutely suppresses melatonin secretion (Drijfhout et al. 1996a).

Understanding of the melatonin suppression response to light in school‐age children, adolescents, and adults is rapidly increasing; however, a large gap in the literature exists in the early childhood years. Findings from several studies examining changes in ocular features across the lifespan have proposed a decrease in circadian photoreception with age. For example cross‐sectional data show that young children have lenses that are more transparent and pupils that are larger than adults (Weale 1985; Yang et al. 2002; Charman 2003). By the age of 45, humans have roughly half the circadian photoreception as a 10‐year‐old due to age‐related yellowing of the crystalline lens and pupillary miosis (Turner and Mainster 2008). Furthermore, one recent study examining pupil size in a controlled moderate bright light environment found that school‐age children had larger pupil diameters than their parents (Higuchi et al. 2014). Together, these data suggest that higher lens transparency and greater pupil size facilitate increased retinal illumination, which likely results in a stronger signal to the SCN in children than adults (Charman 2003; Turner and Mainster 2008). These findings highlight the need for experimental data quantifying the melatonin suppression response in young children. In this study, we address this gap in the literature by examining the magnitude of light‐induced melatonin suppression to evening bright light in preschool‐age children.

## Materials and Methods

### Participants

This study included 10 healthy children ages 3–5 years (seven females; nine Caucasian, one mixed race) aged 4.3 ± 1.1 years (*M *± *SD*). Families were recruited from the Boulder, CO area through posted flyers, a laboratory database of former study participants, and a database of local residents who expressed interest in having their children participate in research studies through the University of Colorado Boulder.

Parents of 14 children completed a telephone screening interview and online surveys to assess inclusion/exclusion criteria. Of these, 11 children were enrolled and 10 completed the study. Exclusion criteria included parental report of any of the following: behavioral/emotional problem, chronic illness, metabolic disorder, serious infectious illness, neurological disorder, or developmental disorder; a reported habitual sleep schedule differing by greater than 2‐h between weekdays and weekends; travel beyond two or more time zones within 2 months of the study; medication use influencing the sleep or circadian systems; family history of a sleep or psychiatric disorder; light sensitizing medication use in the 1 year before the study (reviewed by a physician); or diagnosed visual impairment (e.g., color blindness, impaired pupillary reaction to light). Parents provided written informed consent, as approved by the Institutional Review Board at the University of Colorado Boulder. Families were compensated $150 for completing the study.

### Protocol

**Figure 1:**
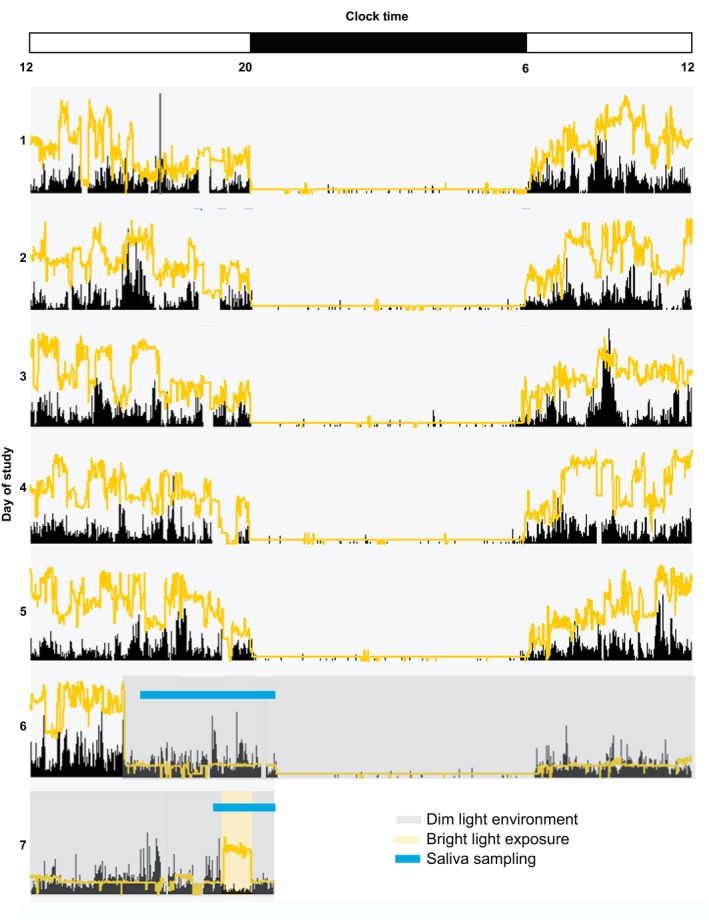
Experimental Protocol. Actogram for a 4‐year‐old participant wearing an Actiwatch Spectrum. Black tick marks represent activity and yellow line represents light exposure in lux (scale 0.1 lux–200,000 lux). Clock hour is indicated on the *x*‐axis and day of study on the *y*‐axis. The dark bar on the x‐axis represents this individual subject's sleep interval (time in bed; 20:00–6:00). On days 1–5, children followed a strict sleep/wake schedule. On day 6, children entered dim light conditions (<15 lux; denoted by gray shading) 1‐h before the start of saliva sampling, where they remained until bright light exposure (~1000 lux; denoted by yellow shading) on day 7. Subjects returned to dim light conditions for 50‐min following the bright light stimulus. Times of saliva sampling are denoted by the blue line on days 6 and 7.

Children participated in a 7‐day protocol (Fig.**** 1) in July–September 2015. On days 1–5, subjects followed a strict sleep/wakefulness schedule and refrained from caffeine and medications in the 96‐h before data collection. Researchers contacted parents daily to confirm sleep schedule compliance and made several in‐home visits to train subjects on providing adequate saliva samples. Subjects wore an actigraph (AW Spectrum, Phillips/Respironics, Pittsburg, PA, USA) throughout the study to obtain an objective measure of sleep. Parents reported their child's sleep with a daily diary (Akacem et al. 2015), which was used to facilitate actigraphy data scoring as detailed in our previous publication (LeBourgeois et al. 2013). For one child, we replaced actigraphic measures with sleep diary data due to noncompliance with wearing the actigraph. Children also wore a pendant light meter (Dimesimeter, Lighting Research Institute, Troy, NY) around their neck during periods of wakefulness for the duration of the study to measure minute‐by‐minute light exposure (lux).

On the afternoon of day 6, children participated in the dim light condition. An in‐home dim light environment of <15 lux was created by covering windows with black plastic and using low wattage bulbs and dimmer switches to manipulate existing light sources. Children entered dim light conditions 1‐h before the first saliva sample. They remained seated for 5‐min and did not eat or drink for 15‐min before each saliva sample. After eating, children chewed on a dry dental cotton roll and/or rinsed their mouth with water to remove any food debris at least 15‐min before the next saliva sample. Saliva samples were collected by having subjects chew on a dry dental cotton roll (Henry Schein Inc., Denver, PA, USA) for ~2‐min to produce ≥2 mL of saliva. Samples were centrifuged (LabEssentials Inc., Monroe, GA, USA), refrigerated on site, and frozen at the laboratory within 1‐h of the end of the assessment (−80°C). Light readings were taken in children's angle of gaze at each saliva sample using a lux meter held approximately 5 cm from the face (Extech Instruments, Spring Hill, FL, USA).

Assays were performed at Solid Phase Inc. (Portland, ME, USA) using radioimmunoassay (ALPCO Diagnostics, Salem, NH, USA) with a functional sensitivity of 0.9 pg/mL and an analytical sensitivity of 0.2 pg/mL. A logit‐log data reduction program was used to calculate melatonin concentrations between 0.0 and 0.5 pg/mL. The intra and interassay coefficients of variation were 4.1% and 6.6%, respectively.

On day 6, the first saliva sample occurred 3‐h and 20‐min before scheduled bedtime. The first five samples on this evening were taken 30‐min apart. During the 1‐h before scheduled bedtime, samples were taken every 20‐min (samples 6, 7, and 8). Samples were taken more frequently during the hour before bedtime in order to provide greater temporal resolution of changes in melatonin levels during the light exposure. Two more samples were taken after scheduled bedtime (30‐min apart; samples 9 and 10). After the last saliva sample on this evening, children were put to bed by their parents. Dim light conditions were maintained the next day.

On day 7, researchers arrived at the subject's home just before scheduled wake time and stayed with the child for the day to ensure that dim light conditions were maintained within the home. Twenty minutes before the start of the light exposure, children provided one saliva sample in dim light (occurred at the same time as sample 5 the previous night). During the hour before habitual bedtime, subjects were exposed to bright light for 1‐h via a “light table” made from a plastic storage bin (Sterilite, Towensend, MA, USA). A light box was placed inside the bin (Phillips Original Bright Light HF3301, Andover, MA, USA; Phillips 55‐Watt Natural 5000K Energy Saver Compact Fluorescent Light Bulb, Andover, MA, USA) and was covered with a neutral density filter with 51.2% transmission (LEE Filters 209 .3ND, Burbank, CA, USA) to attenuate the intensity of the light box and provide ~1000 lux of light at the subject's angle of gaze. The top of the light table was made from a clear sheet of polycarbonate (Sabic Innovative Plastics, Pittsfield, MA, USA). Light measurements were taken at the subject's angle of gaze every 10‐min throughout the light exposure. Children were exposed to an average of 1033 ± 158 lux during the experimental light stimulus. Researchers played with subjects at the light table to ensure their angle of gaze was downwards toward the light source. Activities at the light table included coloring on clear overhead sheets and playing with open magnetic tiles to maximize the subject's light exposure. Saliva samples were obtained 10‐, 30‐, and 50‐min after the start of the light exposure (time anchored to samples 6, 7, and 8 taken on day 6). Following the light exposure, subjects re‐entered dim light conditions (<15 lux) and provided two more saliva samples occurring 20‐ and 50‐min following the light exposure (time anchored to samples 9 and 10 from day 6).

The intensity of the chosen light stimulus was based upon the adult illuminance response curve which dictates ~1000 lux induces a near saturating melatonin suppression response (Zeitzer et al. 2000). This intensity of light is also relatively similar to the average amount of light preschool‐age children living in Colorado are exposed to in the 2‐h before bedtime (710.1 ± 1418.2 lux) (Akacem et al. 2016).

### Data analysis and statistics

Percent melatonin suppression was computed using area under the curve (AUC; trapezoidal method; MATLAB, MathWorks, Natick, MA, USA). Specifically, area under the curve was calculated for the duration of the bright light stimulus (AUC_BL_) and compared to AUC at the same clock times in the dim light condition on day 6 (AUC_DL_). Melatonin suppression was computed using the following equation: [1‐AUC_BL_/AUC_DL_] × 100 (Gooley et al. 2011). Melatonin levels were compared between conditions using a paired samples *t*‐test with a Bonferroni correction (*P* ≤ 0.008; SPSS Statistics, IBM Corp., Armonk, NY, USA). All paired t‐tests were two‐tailed. Dim light melatonin onset (DLMO) was computed as the clock time salivary melatonin levels passed 4 pg/mL in the dim light condition (Deacon and Arendt 1994; Carskadon et al. 1997). One child's melatonin levels did not pass this threshold, and thus, we were unable to determine the timing of DLMO for this subject. Hence, average DLMO timing and the phase angle between DLMO and the start of the light exposure presented here reflects a sample size of 9 participants.

## Results

**Figure 2:**
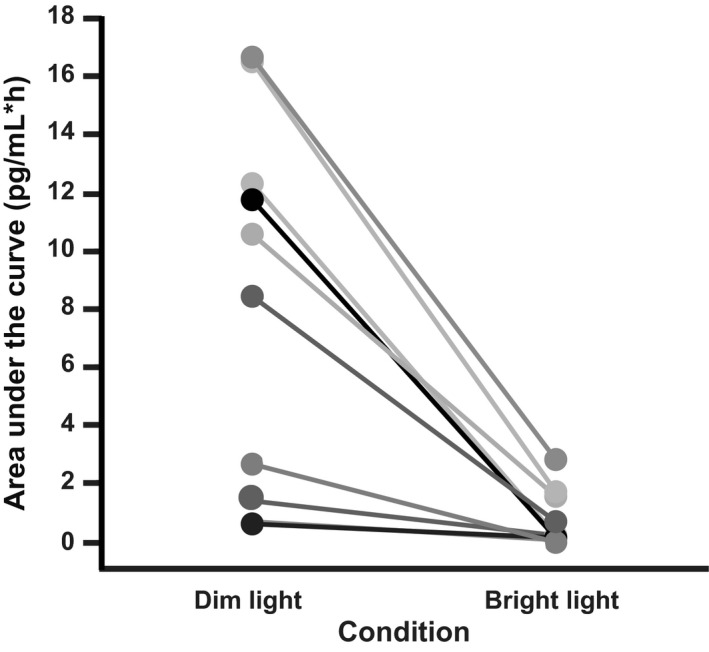
Melatonin Area Under the Curve (AUC) for Dim and Bright Light Conditions. Melatonin AUC is shown for the duration of the bright light stimulus (bright light condition) and at the same relative clock times in the dim light condition for each individual subject. AUC was lower in the bright light condition for all subjects.

**Figure 3:**
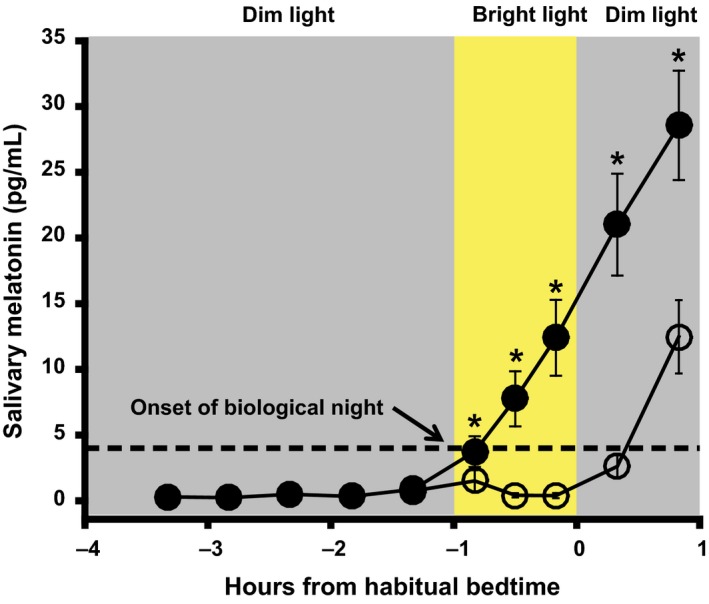
Melatonin Suppression. Average melatonin profile in the dim light exposure night and bright light exposure night. Error bars represent standard error. Mean melatonin suppression was 87.6 ± 10.0% (M±SD). Melatonin levels in samples taken 20‐min before the start of bright light exposure were not significantly different between conditions (*P* = 0.65). Melatonin levels were significantly lower in samples taken at 10‐, 30,‐ and 50‐min after the start of bright light exposure in the bright light versus the dim light condition (*P ≤ 0.008; Bonferroni correction). Compared to the dim light condition, melatonin levels in the bright light condition remained lower 20‐ and 50‐min after children returned to dim light.

Children were exposed to an average 48,545.04 ± 107,415.40 lux per day in the 5 days preceding the experimental protocol. Descriptive statistics on sleep and circadian parameters are reported in Table**** 1. Average bedtime during the 5 days before the start of the experimental protocol was 20:27 ± 00:17. The timing of the dim light melatonin onset (DLMO), a marker of the beginning of the biological night, was 19:47 ± 00:34 during the dim light condition (*n* = 9; see Data Analysis and Statistics). The time interval between the start of the bright light stimulus and DLMO ranged from ~70‐min before to ~12‐min after the DLMO (20.1 ± 29.1 min before DLMO). Melatonin levels 20‐min before the start of the light exposure were not significantly different between conditions (*P *=* *0.65). Average melatonin suppression across the light exposure was 87.6 ± 10.0%. Area under the curve during the light exposure (AUC_BL_) and at the same relative clock times during the dim light condition (AUC_DL_) are plotted for each subject in Figure 2. Melatonin levels at 10‐min (*P *=* *0.008, *d *=* *0.70), 30‐min (*P *=* *0.006, *d *=* *1.56), and 50‐min after the start of the light exposure (*P *=* *0.002, *d *=* *1.86) were significantly lower than levels at the same clock times in the dim light condition (Fig.**** 3). Melatonin levels remained significantly lower at 20‐min (*P *<* *0.001, *d *=* *2.07) and 50‐min following the end of the bright light exposure condition (*P *<* *0.001, *d *=* *1.43) compared to the dim light condition (Fig.**** 3). Furthermore, at 50‐min after light exposure, melatonin levels did not return to 50% of those observed in the dim light condition for 7/10 children.

**Table 1:** Descriptive statistics for sleep and circadian variables

	*M*	*SD*
Sleep variables (*n* = 10)		
Bedtime	20:27	0:17
Sleep onset time	20:54	0:28
Midsleep time	1:50	0:35
Sleep end time	6:47	0:47
Wake time	6:56	0:45
Sleep onset latency (min)	26.5	13.4
Circadian variables (*n* = 10)		
Dim light melatonin onset time	19:46	0:34
Bedtime phase angle (min)	40.2	30.4
Sleep onset phase angle (min)	64.0	33.6
Midsleep phase angle (min)	356.8	38.7
Sleep offset phase angle (min)	649.7	47.1
Wake time phase angle (min)	658.6	42.4
Bright light exposure phase angle (min)	39.9	29.1

## Discussion

To our knowledge, this study is the first to quantify the melatonin suppression response to evening light exposure in healthy 3‐ to 5‐year‐old children. A combination of well‐controlled procedures minimizing sources of error variance, a repeated‐measures design, and large effect sizes led to several significant findings: (a) 1‐h of bright light exposure before bedtime suppresses melatonin by ~88%; (b) the effects of evening bright light exposure on melatonin levels persisted at least 50‐min following termination of the light stimulus; and (c) melatonin levels remained attenuated and did not return to 50% of those observed in the dim light condition following the bright light exposure for the majority of the children.

Findings from this study mark an important step in addressing a gap in understanding the melatonin suppression response across the lifespan. To date, published data indicate a trend for greater sensitivity to the melatonin suppression effects of light early in life and a decrease in sensitivity with age. Up until now, school‐age children were the youngest age at which the melatonin suppression effects of light have been investigated (Higuchi et al. 2014). Findings from this study indicated that school‐age children were almost twice as sensitive to the melatonin suppression effects of evening moderate bright light exposure (~580 lux) than their parents (Higuchi et al. 2014). Additionally, based upon an objective measure of pubertal development (Tanner 1962), Crowley and colleagues found that that prepubertal adolescents (Tanner stage 1–3) were more sensitive to the suppressive effects of evening light on melatonin than postpubertal adolescents (Tanner stage 4–5) (Crowley et al. 2015). Another study comparing melatonin suppression in response to short‐wavelength light found that younger adults experienced greater melatonin suppression than older adults (Herljevic et al. 2005). Although our data contribute to an understanding of age‐related changes in circadian sensitivity to light, a longitudinal within‐subject design study is necessary to fully understand changes in the melatonin suppression response to light across the lifespan.

Due to differences in methodology, including variable prior light history and timing of the experimental light exposure, comparisons between our findings and others examining the melatonin suppression response must be made with caution. Nonetheless, intensity and duration response curves to light in adults provide a context for our findings. The duration response curve to light shows that a 1‐h light stimulus of 10,000 lux, 10 times the intensity of light used in this study, suppressed melatonin by 39 ± 19% (Chang et al. 2012). The intensity response curve shows ~93% suppression in response to 6.5 h of 1000 lux (Zeitzer et al. 2000). Considering the proposed differences in circadian photoreception with age (Turner and Mainster 2008), intensity and duration response curves to light in preschool‐age children are necessary to offer a comprehensive understanding of the dynamics of the melatonin suppression response in this age group.

Given the role of the circadian clock and melatonin in the regulation and promotion of sleep, our findings may have important implications for understanding the etiology of evening sleep problems in early childhood. Approximately 30% of young children experience difficulties transitioning from wakefulness to sleep, including bedtime resistance and sleep onset delay (Beltramini and Hertzig 1983; Lozoff et al. 1985; Bruni et al. 2000). It is well‐known that melatonin is responsible for preparing the body for sleep (Cajochen et al. 2003). Thus, the robust and sustained suppression (at least 50‐min) we observed in response to evening bright light exposure before bedtime may impair young children's success in falling asleep. Our findings may be especially important in the context of an abrupt transition from relatively bright indoor light before bedtime to dim/dark conditions at lights‐off that may be part of children's bedtime routine. Studies promoting an understanding of both the relationship between light‐induced melatonin suppression and nighttime settling as well as the dynamics and duration of the melatonin recovery process following light exposure in young children are important future directions of this work.

In addition to the acute light‐induced suppression of melatonin, a delay in circadian timing may be another pathway by which evening light exposure can contribute to evening sleep problems in young children. Although this study was not designed to assess changes in circadian timing (phase shifts) in response to an experimental light stimulus, this represents an area of rich investigation. Currently, phase response curves to light that predict both the magnitude and direction of phase shifts in response to light presented at various biological times have only been published for adults (Khalsa et al. 2003) and adolescents (Crowley and Eastman 2017). The magnitude of the circadian response to light may differ in young children and therefore understanding the phase shifting effects of light in this age group is necessary to support age‐based recommendations for evening light exposure.

Beyond promoting sleep, the pineal hormone melatonin plays an important role in overall health and normal physiological functioning (Pandi‐Perumal et al. 2008). Melatonin is the body's internal signal of the biological night and has been implicated in a variety of essential physiological processes, including thermoregulation (Saarela and Reiter 1994), blood pressure (Simko and Paulis 2007), and glucose homeostasis (la Fleur et al. 2001; Owino et al. 2016). Additionally, melatonin functions as an antioxidant in the body by interacting with intracellular components (Reiter et al. 1999). An established literature in adults demonstrates that inappropriately timed light exposure during the biological night can suppress melatonin and disrupt normal physiological functioning that may contribute to a variety of negative health outcomes including obesity (Fonken et al. 2010; Tan et al. 2011), diabetes (Qian et al. 2013) and cancer (Schernhammer and Schulmeister 2004). Because the early childhood years are a sensitive and vulnerable window in human development (Center on the Developing Child, 2010), future research should investigate the role of evening light exposure in health and disease in early life.

To contextualize the brightness of the experimental light stimulus used in this study, typical indoor lighting is <200 lux (Wright et al. 2013), while bright indoor lighting can range from 300 to 1000 lux (Boubekri et al. 2014). Although the 1000 lux stimulus administered in this study would be similar to a brightly lit environment, it is not comparable to the intensity of light emitted from electronic devices (~30–50 lux; (Chang et al. 2015)). Still, our findings of robust melatonin suppression in this age group call for an understanding of the effects of light‐emitting electronic devices on the circadian physiology of young children. The amount of time young children spend using mobile electronic devices has tripled in recent years (Common Sense Media, 2011), and 90% of parents report that their children use electronic media devices before the age of 2 years (American Academy of Pediatrics, 2011). Often electronic device use in this young age group occurs in the hour before bedtime and is therefore, incorporated into a child's bedtime routine (Vandewater et al. 2007). Findings from our laboratory indicate that the natural increase in endogenous melatonin in well‐controlled dim light conditions occurs ~50‐min before bedtime (i.e., bedtime phase angle; interval between melatonin onset and bedtime) in young children (LeBourgeois et al. 2013). Thus, the 1‐h before bedtime may represent a window during which the circadian system is sensitive to perturbations (i.e., suppression of melatonin and phase delays) (Khalsa et al. 2003). Recently published data demonstrate that adults are sensitive to the effects of electronic media use in the 1‐h before bedtime (Chang et al. 2015). Specifically, electronic device use before bedtime suppressed melatonin, delayed the timing of melatonin onset, increased sleep onset latency by 10‐min, and negatively affected next day alertness. Considering that the screens of most electronic devices peak in the short wavelengths (Gringras et al. 2015) and lens transparency differences across age are most prominent at shorter wavelengths (Charman 2003), we expect that young children are more sensitive to the effects of light emitted from these sources. The almost ubiquitous nature of electronic media use in this young age group supports the critical need for studies assessing the effect of evening electronic media use on melatonin levels, circadian timing, and subsequent sleep and alertness early childhood (LeBourgeois et al. 2017).

Although we employed a well‐controlled experimental protocol to test our hypothesis, this study is not without limitations. First, we enrolled a relatively small cross‐sectional sample of children from a narrow age range. Additional research with a larger sample of children is necessary to establish a more accurate estimate of light‐induced melatonin suppression effects, and longitudinal data are critical to determine whether such a response decreases with age. Second, the experimental light stimulus was anchored to the child's bedtime and not intrinsic circadian timing. Thus, subjects were exposed to the bright light stimulus at various circadian phases, suggesting that future research should consider individual circadian timing when delivering a light stimulus. Additionally, subjects remained in <15 lux conditions preceding the experimental light stimulus in order to control for the effects of prior light history on the subsequent circadian response to light. This dim light environment may have sensitized young children to the light stimulus and influenced the magnitude of melatonin suppression observed (Smith et al. 2004; Chang et al. 2011). Lastly, this study included both napping and non‐napping young children, who based upon the 2‐process model of sleep regulation (Werth et al. 1996) and our recent findings (Lassonde et al. 2016), likely had different levels of accumulated sleep pressure at bedtime. For this reason, we were not able to assess the effects of evening bright light exposure on subsequent sleep parameters (e.g., sleep onset latency, bedtime resistance), suggesting the need for studies of independent samples of napping and non‐napping children.

## Perspectives and Significance

In this well‐controlled innovative study, we found a robust and sustained response of the circadian system of young children to evening bright light exposure. Together, these data and our approach represent a first step in understanding the dynamics of the circadian response to light in early childhood and suggest that evening light exposure may increase risk for evening sleep disturbances in preschool‐age children. This work also serves as a foundation for future studies aimed at understanding the phase shifting response to varying intensities, durations, and/or spectrums of light in young children. Such experimental data are needed to make informed recommendations to parents and health‐care professions and may have important clinical relevance in preventing the development of late sleep timing in the early years of life.

## Conflicts of Interest

MKL: Speaker honorarium from Integrated Listening Systems. KPW: Funding from Philips Inc., Torvec Inc., and PAC‐12; Consulting fees or served as a paid member of scientific advisory boards for NIH, Torvec Inc.; Speaker honorarium from American College of Chest Physicians, The Obesity Society, Obesity Medicine Association.

## Data Accessibility
